# Moving on up: Vertical distribution shifts in rocky reef fish species during climate‐driven decline in dissolved oxygen from 1995 to 2009

**DOI:** 10.1111/gcb.15821

**Published:** 2021-09-16

**Authors:** Erin Meyer‐Gutbrod, Li Kui, Robert Miller, Mary Nishimoto, Linda Snook, Milton Love

**Affiliations:** ^1^ School of the Earth, Ocean and Environment University of South Carolina Columbia SC USA; ^2^ Marine Science Institute University of California Santa Barbara CA USA

**Keywords:** climate change, deoxygenation, distribution, hypoxia, marine ecosystem, oxygen minimum zone, rockfish, rocky reef

## Abstract

Anthropogenic climate change has resulted in warming temperatures and reduced oxygen concentrations in the global oceans. Much remains unknown on the impacts of reduced oxygen concentrations on the biology and distribution of marine fishes. In the Southern California Channel Islands, visual fish surveys were conducted frequently in a manned submersible at three rocky reefs between 1995 and 2009. This area is characterized by a steep bathymetric gradient, with the surveyed sites Anacapa Passage, Footprint and Piggy Bank corresponding to depths near 50, 150 and 300 m. Poisson models were developed for each fish species observed consistently in this network of rocky reefs to determine the impact of depth and year on fish peak distribution. The interaction of depth and year was significant in 23 fish types, with 19 of the modelled peak distributions shifting to a shallower depth over the surveyed time period. Across the 23 fish types, the peak distribution shoaled at an average rate of 8.7 m of vertical depth per decade. Many of the species included in the study, including California sheephead, copper rockfish and blue rockfish, are targeted by commercial and recreational fisheries. CalCOFI hydrographic samples are used to demonstrate significant declines in dissolved oxygen at stations near the survey sites which are forced by a combination of natural multidecadal oscillations and anthropogenic climate change. This study demonstrates in situ fish depth distribution shifts over a 15‐year period concurrent with oxygen decline. Climate‐driven distribution shifts in response to deoxygenation have important implications for fisheries management, including habitat reduction, habitat compression, novel trophic dynamics and reduced body condition. Continued efforts to predict the formation and severity of hypoxic zones and their impact on fisheries dynamics will be essential to guiding effective placement of protected areas and fisheries regulations.

## Graphical abstract

This study examined changes in species distributions along a steep bathymetric gradient from 1995 to 2009 to determine whether declining concentrations of dissolved oxygen has triggered redistributions in marine ecosystems. One third of fish species observed on Southern California rocky reefs exhibited a significant shift in peak distribution to shallower habitat concurrent with shoaling hypoxic waters. As anthropogenic warming and ocean deoxygenation intensify, the rapid redistribution of marine species to more suitable habitats and the extirpation of populations that are unable to relocate will have unprecedented consequences on marine ecosystem structure and the provisioning of ecosystem resources

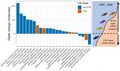

## 1 INTRODUCTION

Greenhouse gas emissions have driven global increases in atmospheric and ocean temperatures, which enhance ocean stratification. As the ocean surface layer becomes more buoyant, transport of highly oxygenated surface waters into the ocean interior is reduced (Keeling & Garcia, 2002). Increases in seawater temperature also reduce oxygen solubility (Schmidtko et al., 2017). Marked declines in dissolved oxygen (DO) concentrations and shoaling of the oxygen minimum zone (OMZ) have been observed globally since 1960 (Stramma et al., 2008). These anthropogenic processes are superimposed on natural multidecadal oscillations (Deutsch et al., 2011; Stramma et al., 2019), seasonal patterns (Boyer et al., 1999; Connolly et al., 2010) and storm impacts (Van Dolah & Anderson, 1991; Xu et al., 2019) on ocean oxygen content.

The ecological impacts of reduced ocean oxygen concentrations include altered microbial processes and metabolic rates, changes in predator–prey dynamics and lateral and vertical distribution shifts in marine organisms (Deutsch et al., 2015; Gilly et al., 2013). Hypoxia disproportionately impacts large taxa, including crustaceans, echinoderms and fish, and is associated with decreased fecundity, habitat reductions and a loss of diversity (Levin et al., 2009; McClatchie et al., 2010; Sato et al., 2018; Stramma et al., 2012). Exposure to hypoxic conditions over a short period of time can often be tolerated by temporary metabolic reductions (i.e. Chew et al., 1990); however exposure over long time periods can lead to growth restrictions, increased risk of predation and mortality (van den Thillart et al., 1994). The impact of low DO concentrations is highly variable between fish species (Davis, 1975; Gray et al., 2002). In a broad review of fish response to hypoxia, Gray et al., (2002) found that many actively swimming fish exhibit growth restrictions at concentrations of 4.2 ml/L and metabolic rates decreased at 2.8 ml/L for benthic fish; mortality can occur for many species at 1.4 ml/L. The observed and forecasted expansion of hypoxic waters have the potential to impact commercial fishing productivity and create regulatory challenges across political boundaries (e.g. Cheung et al., 2012).

The upper 3000 m of the Northeast Pacific has lost over 15% of its oxygen over the last 60 years, with the OMZ expanding at a rate of 3.0 m/year (Ross et al., 2020). Hydrographic data from the California Cooperative Oceanic Fisheries Investigations (CalCOFI) program demonstrate DO declines and OMZ shoaling beginning in the 1980s in the southern California Current System (CCS) (Bograd et al., 2008). In this time period, the hypoxic boundary has shoaled to depths as shallow as 90 m in parts of Santa Barbara Channel and areas off Point Conception (Bograd et al., 2008). This expansion and shoaling of the OMZ has the potential to impact fish populations and communities through community reorganization and habitat compression. Previous studies provide insight on the effects of low DO on fish survival, fitness and distribution in the productive California Coastal Current (e.g. Chan et al., 2008; Davis et al., 2018; Flannery, 2018; Gallo, Hardy, et al., 2020; Keller et al., 2017; McClatchie et al., 2010).

Laboratory experiments comparing fish behaviour and metabolic rates between normal and low DO treatments provide a basis to predict fish response to changes in their native habitat. For example, juvenile rockfish species (including gopher rockfish, *Sebastes caratus*; copper rockfish, *S*. *caurinus*; and black‐and‐yellow rockfish, *S*. *chrysomelas*) from central California that were exposed to hypoxic conditions (DO concentration of 3.15 ml/L) exhibited increased metabolic costs, exploration behaviour and predation mortality compared to normoxic controls (Davis et al., 2018). The swimming performance of juvenile copper rockfish (*Sebastes caurinus*) and black rockfish (*Sebastes melanops*) from northern California decreased in hypoxic conditions (DO concentration of 2.8 ml/L and 1.4 ml/L, Flannery, 2018). In laboratory experiments with juvenile rockfish collected from Central California exposed to hypoxic conditions, copper rockfish exhibited behavioural changes such as reduced escape time, and blue rockfish (*Sebastes mystinus*) experienced elevated mortality rates (Mattiasen et al., 2020). These studies indicate that declining DO may lead to distributional shifts in California rockfish populations, or cause a decrease in survival and fecundity in persistent populations.

Oxygen concentrations have been repeatedly identified as a significant predictor in pelagic and demersal fish distribution (Gallo & Levin, 2016; Netburn & Koslow, 2015). In a study by Gallo, Beckwith, et al., (2020), a remotely operated vehicle (ROV) was used to survey benthic fish communities in the Gulf of California at depths ranging from 200 m to 1400 m. Oxygen level was the best predictor of fish community composition and diversity, and declines in oxygen predicted by a global climate model are expected to drive a reduction in diversity by 2081–2100 (Gallo, Beckwith, et al., 2020). Observations from an autonomous lander at depths from 100–400 m off the coast of San Diego indicate that benthic communities transitioned from fish dominated to invertebrate dominated along a declining oxygen gradient (Gallo, Hardy, et al., 2020). West Coast Groundfish Bottom Trawl surveys conducted within a known hypoxic zone off the coast of Oregon show significantly lower weight to length ratios in five of six groundfish species in low DO regions (<1 ml/L) relative to moderate regions (>1 ml/L, Keller et al., 2010). A temporary anoxic event in the California Current large marine ecosystem was accompanied by the near‐complete mortality or abandonment of the anoxic zone by rocky reef macroscopic benthic invertebrates and fish (Chan et al., 2008).

Changes have also been detected in fishery productivity between normal and low DO environments. US West Coast Groundfish Bottom Trawl catch per unit effort was positively associated with DO for 19 of 34 groundfish species in hypoxic (DO <1.43 ml/L) or severely hypoxic (DO <0.5 ml/L) environments (Keller et al., 2017). Total catch per unit effort and species richness were also positively associated with DO concentrations within hypoxic waters (Keller et al., 2010, 2015, 2017). Periodic declines in ichthyoplankton abundance in the southern California Current corresponding to low oxygen observed during CalCOFI surveys from 1951 to 2008 indicate that hypoxia may also reduce mesopelagic fish recruitment (Koslow et al., 2011), although not for all species (Koslow et al., 2019). During the summer months from 1950 to 2007, hypoxic conditions (DO <1.5 ml/L) were detected in 37% of the rockfish habitats in the Cowcod Conservation Area (McClatchie et al., 2010). Although hypoxic conditions in this conservation area had the potential to slow the recovery of the overfished cowcod (*Sebastes levis*) stock, the population has since recovered steadily and the stock was declared rebuilt by the Pacific Fishery Management Council in 2019 (Dick & He, 2019).

While previous experimental and field studies have compared fish fitness and distribution between normoxic and hypoxic waters off the California coast and throughout the Northeast Pacific, in situ changes in fish communities over time in response to declining DO have not been examined. In this study, we conducted visual fish transect surveys on rocky reefs off the California Channel Islands over a 15‐year period. The surveyed reefs fall along a steep bathymetric gradient, making this an ideal site for the observation and quantification of species‐specific changes in depth distribution over time. Adjacent CalCOFI stations characterized the depth‐stratified temperature and oxygen concentrations impinging on these reefs throughout the time series. We hypothesized that depth‐dependent declines in DO over time would correspond to shoaling of the peak distribution of fish species along this reef network. Identifying ongoing and forthcoming changes in the distribution and habitat loss of demersal fish is critical in guiding the management of associated commercial and recreational fisheries and preparing for novel fishery dynamics resulting from habitat compression due to expanding OMZs.

## 2 METHODS

### 2.1 Fish surveys

**FIGURE 1:**
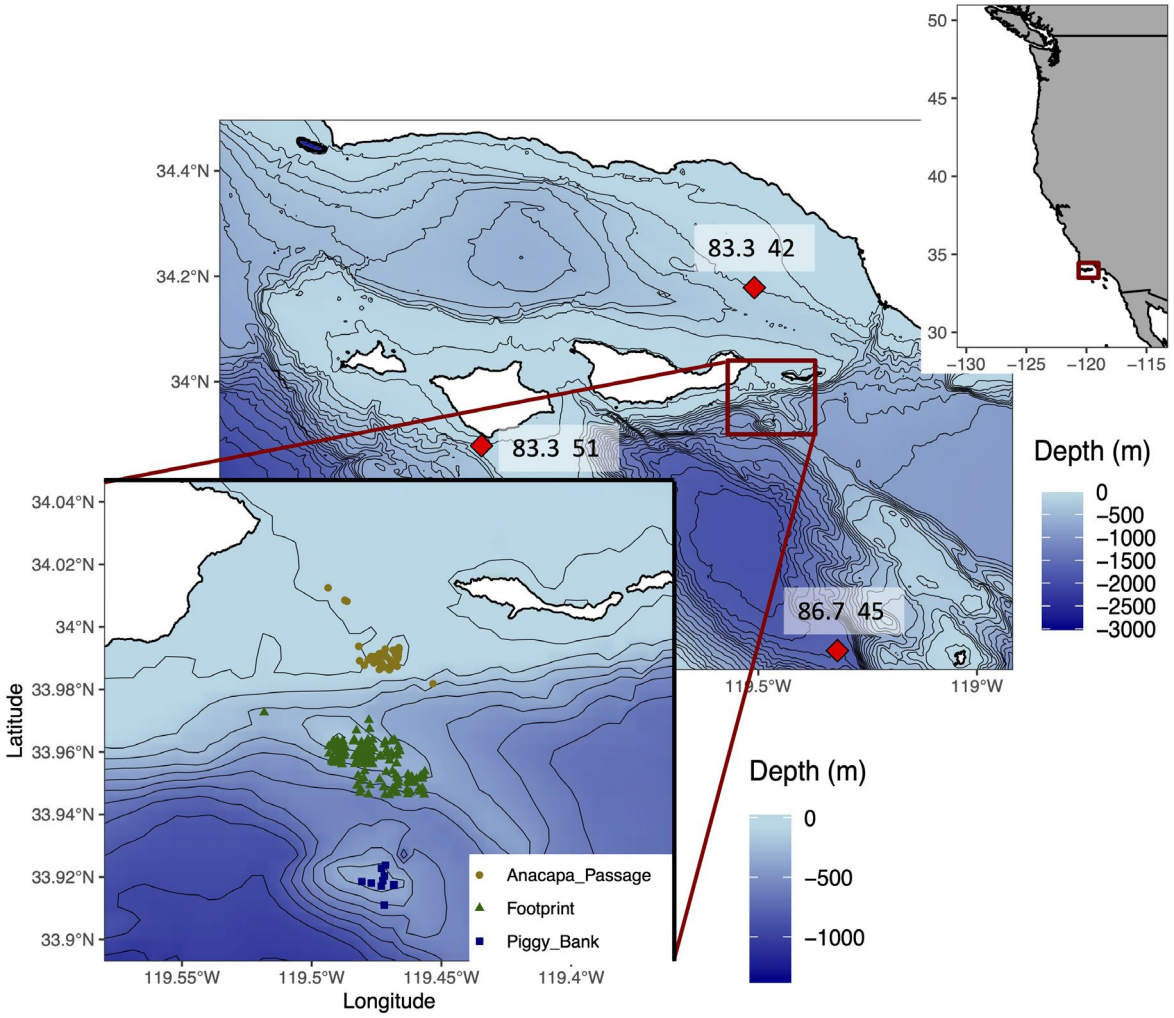
Maps show the US West Coast (top map), the Santa Barbara Channel and CalCOFI stations (centre map) and the fish survey locations at three natural reefs (bottom map). The red box in the US West Coast (top map) indicates the boundaries of the Santa Barbara Channel map, and the red box in the Santa Barbara Channel (centre map) indicates the boundaries of the zoomed in map between Santa Cruz and Anacapa islands. Red diamonds indicate the locations of CalCOFI line 83.3 station 42, line 86.7 station 45 and line 83.3 station 51. Circle, triangle and square shapes indicate the jittered locations of individual survey transects (gold circles = Anacapa Passage, green triangles = Footprint and blue squares = Piggy Bank). Contour lines are drawn at 50‐m depth intervals

**FIGURE 2:**
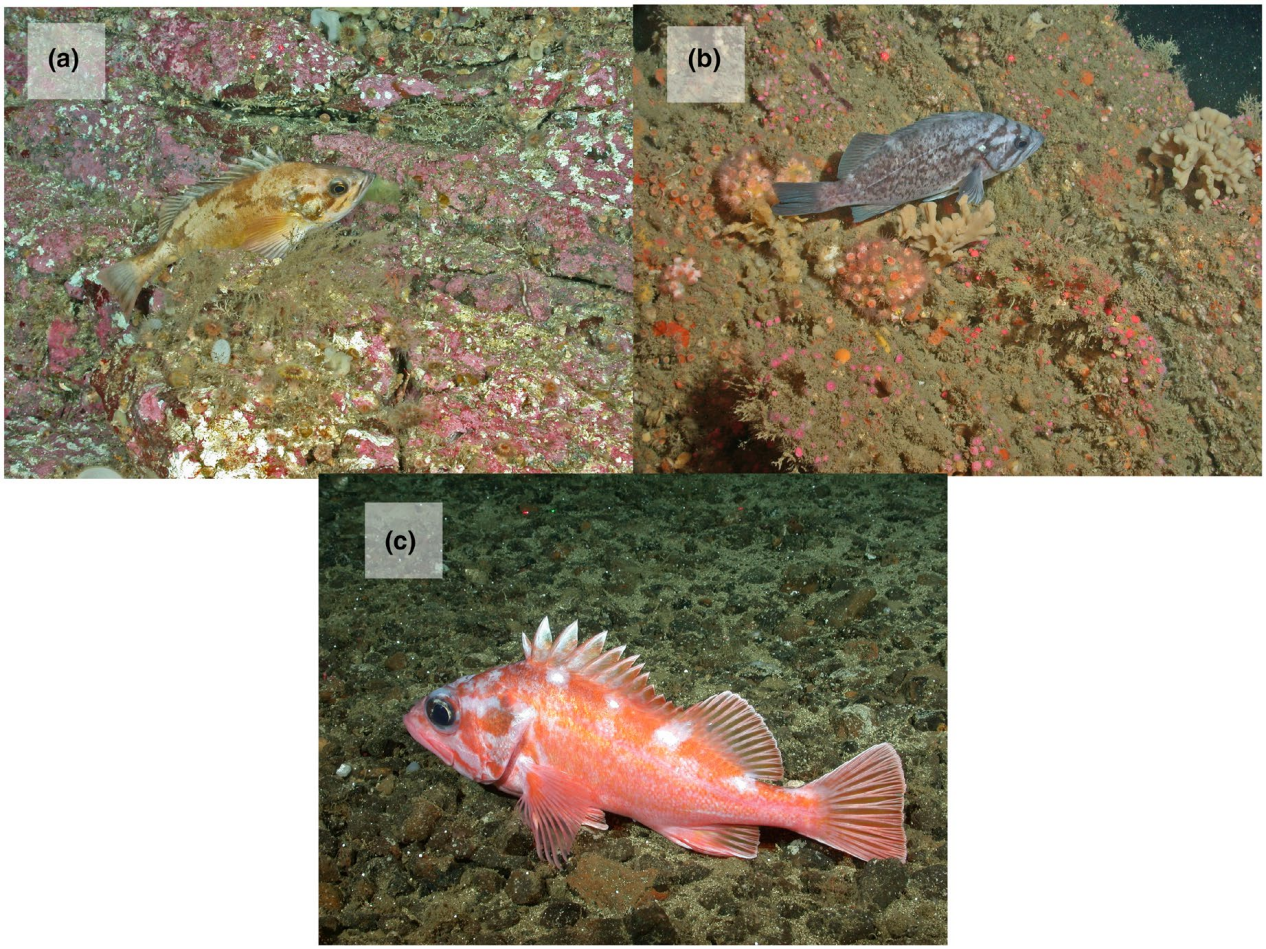
Photographs of a) squarespot rockfish (*Sebastes hopkinsi*), b) blue rockfish (*Sebastes mystinus*) and c) pinkrose rockfish (*Sebastes simulator*) on rocky reefs in the Santa Barbara Channel. Credit: Southwest Fisheries Science Center ROV dive team

Fish were surveyed at three natural rocky reefs located between Santa Cruz Island and Anacapa Island in the Santa Barbara Channel of California (Figure 1, Love et al., 2017). The reefs fall along a steep bathymetric gradient along the edge of the Santa Cruz Basin, and the surveyed depths span a range of 44–365 m. Mean depth of the surveys conducted in the Anacapa Passage, the shallowest and northernmost reef, Footprint, the mid‐depth reef, and Piggy Bank, the deepest and southernmost reef, were 49, 168 and 298 m respectively. The northern edge of Anacapa Passage and the southern edge of Piggy Bank are separated by only 11.2 km, potentially facilitating movement of fish between the three reefs. Fish surveys were conducted between 1995 and 2009. Surveys were not conducted in 1996 or 1997, and surveys from 2002 were excluded due to low survey effort that year. Surveys occurred during daylight hours in the months of September, October and November. Seasonal timing of fish surveys are shown in Figure S1. Fish were visually censused from the 4.6 m long, two‐person *Delta* research submersible, operated by Delta Oceanographics of Oxnard, California. Belt transects with a 2 m width, 2 m height and median transect length of 397 m were conducted at a speed of ~0.5 knots along the seafloor over rocky habitats. Observers counted and identified fish to the lowest taxonomic category possible, typically species (Figure 2). The total length of each individual fish was estimated to the nearest centimetre, with assistance from a pair of parallel lasers mounted 20 cm apart for visual reference. Surveys were recorded with an externally mounted video camera, and footage was reviewed later for accuracy. Additional details of the methodology are found in the study by Love et al., (2009).

### 2.2 Oceanographic data

Oxygen concentration data were pulled from the California Cooperative Oceanic Fisheries Investigations program (CalCOFI) hydrographic bottle data set. Seawater measurements are derived from discrete bottle sample depths collected on CTD/rosette casts during quarterly CalCOFI cruises at 66 stations. While oxygen concentrations are primarily derived from bottle samples, concurrent CTD sensor measurements may be used for corrections and interpolations. Detailed information about the CalCOFI program and methodologies can be found in CalCOFI cruise data reports (e.g. Scripps Institution of Oceanography, 2019). Data were downloaded directly through the CalCOFI website (https://calcofi.org/ccdata/database.html).

Three CalCOFI hydrographic CTD/rosette stations were identified near the reefs to represent local changes in oxygen concentration: CalCOFI line 83.3 station 42, line 86.7 station 45 and line 83.3 station 51 (Figure 1). Estimated bottom depths were as follows for each station: 134 m at line 83.3 station 42, 102 m at line 83.3 station 51 and 1644 m at line 86.7 station 45. For this study, oxygen concentrations were analysed at discrete 50‐m depth intervals ranging from 50 to 300 m. All samples collected within 5 m of a given depth interval were analysed. For example, the 50‐m depth interval used in this analysis consists of samples collected at depths between 45.0 m and 55.0 m.

### 2.3 Analysis

Changes in oxygen concentration at the three CalCOFI stations from 1990 to 2019 were examined using a linear model dependent on year and depth. Two additional models were constructed to assess the changes in temperature and salinity at the three CalCOFI stations over the same time period. A more complex linear model examining change in oxygen concentration over time, including the interaction of year and depth category, was also tested. The 50‐m depth intervals were treated as categorical variables. Only data collected in the months of September, October and November were included in the analysis, corresponding to the months that fish surveys were conducted. Seasonal timing of CalCOFI cruises used in this study are indicated in Figure S1. An additional model of the rate of change in oxygen concentration over time was constructed over the restricted time period 1995–2009 to demonstrate the decline observed during the fish sampling period.

To determine the impacts of depth and time on fish abundance, fish were first separated by taxon and life‐history status. Life history was divided into two categories: young of the year (YOY) and non‐YOY. YOY were defined for each taxon as individuals with a total length less than the average length at 1 year, as predicted by the von Bertalanffy growth function using taxon‐specific growth parameters following Claisse et al. (2014) (Table S1).

Separate models were developed for each unique combination of taxon and life history status that was observed during at least 6 years (half of the study period), and had observation years spanning at least a decade. Fish abundance was modelled using Poisson regression as follows:

$$N_{t,s}∼D+D^{2}+Y+D*Y+\text{offset}\left(\log L\right)$$

where *N* is the number of fish within a specific taxon, *t*, and life‐history stage, *s*, counted on a transect; *D* is the mean depth of the transect; *Y* is the year of the survey; D*Y is the interaction between depth and year; and *L* is the transect length. The depth squared term was added so that fish count estimates were not forced to monotonically increase or decrease across the large depth range observed in this study. This allows the models more flexibility to define the relationship between fish abundance and depth, creating a more realistic fit for species that may have a peak in distribution in this range. All Poisson models were tested for overdispersion using the AER library in R with alpha set at *α* = 0.05 (Kleiber & Zeileis, 2008; R Core Team, 2019). When overdispersion was found, a quasi‐Poisson model was run in place of a Poisson regression.

Model predictions were generated for each survey year across the range of depths that a species was observed. The interaction between depth and year provides information on whether the modelled fish type is moving deeper or shallower over time. We define the annual peak distribution as the depth of maximum predicted fish abundance for a given survey year calculated from the model coefficients. Cases were excluded if a modelled peak in distribution across the three reefs did not occur within the taxon's observed depth range during any of the years within our time series.

The model‐estimated change in the depth of peak distribution over time was calculated for each fish type. This depth change is defined as the difference between the peak depth distribution of the fish type in its first observed year and its final observed year, divided by the span of observation years. This method may result in an underestimate of the change in peak distribution depth because our surveys were limited to depths between 44 m and 365 m. Due to this limitation, we were not able to observe or model peaks in fish densities that may have moved shallower than 44 m over the 15‐year observation span.

## 3 RESULTS

**FIGURE 3:**
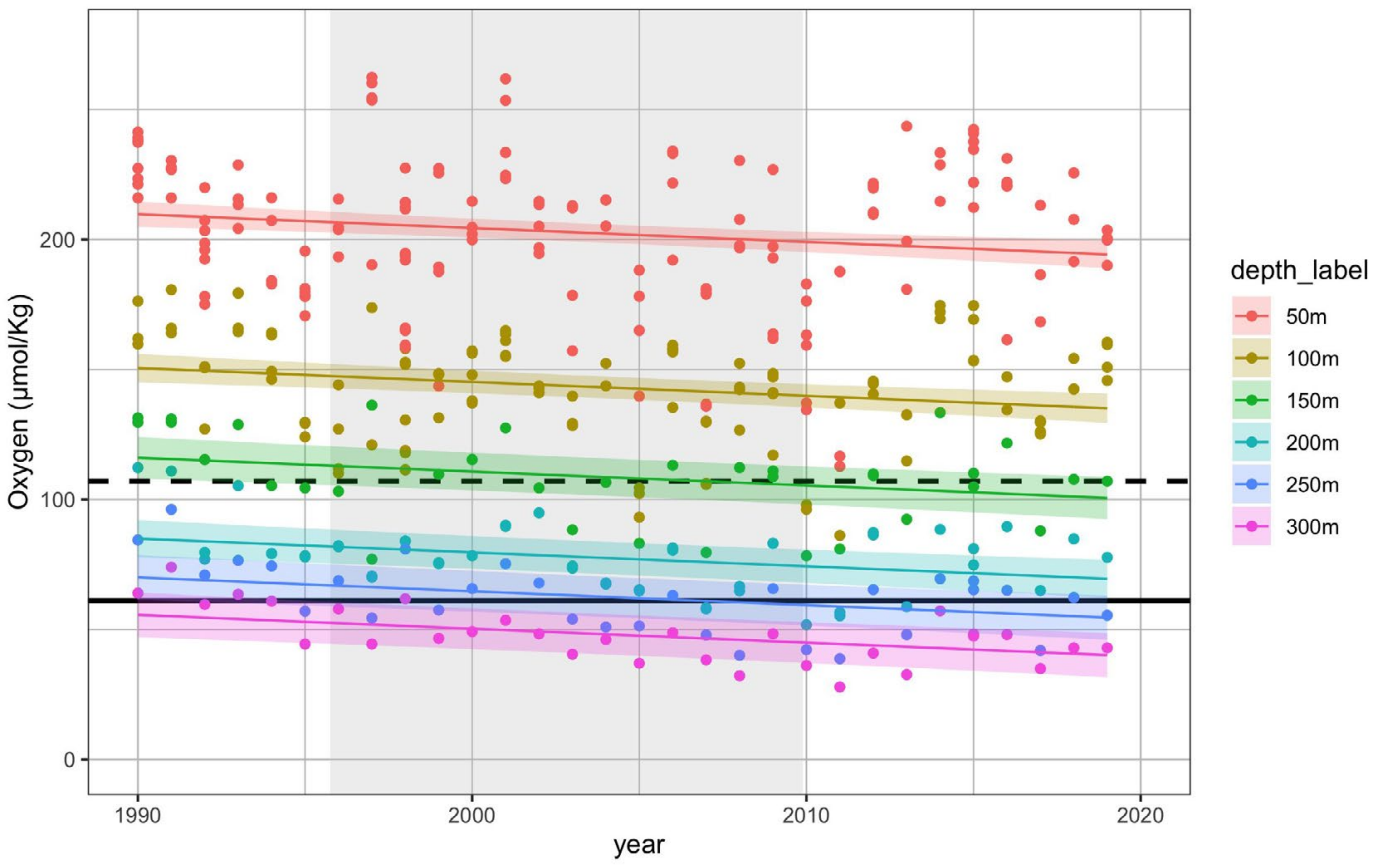
Oxygen concentrations in September, October and November at CalCOFI line 83.3 station 42, line 86.7 station 45 and line 83.3 station 51 at depth bins ranging from 50 to 300 m over the period 1990 to 2019. Grey shading indicates the time period when the ROV fish surveys took place, from fall 1995 to fall 2009. Sampling depth is denoted by colour. Points indicate oxygen measurements and lines represent linear regression predictions of oxygen concentration as a function of year and depth (Table 1); shading around the regression lines represents the 95% confidence intervals for each depth category. Mild hypoxia is defined by the black dashed line at 107 μmol O_2_/kg (~2.45 ml/L), and hypoxia is defined by the black solid line at 61 μmol O_2_/kg (~1.4 ml/L)

The analysis of oxygen concentration data at three CalCOFI stations revealed a significant decline in oxygen over the time period 1990–2019 (*p* < 0.001; Figure 3, Table 1). Deeper depths were characterized by lower oxygen concentrations across the range of depths included in this study. The results from the linear model examining oxygen as a function of year and depth interval indicate that oxygen concentration in this region decreased at a rate of 0.53 μmol O_2_/kg per year. This simple model was a better fit than the more complex model which included the interaction between year and depth category (ΔAIC = 8.75), indicating that the decline in oxygen over time was relatively consistent between depth categories. The same linear oxygen concentration model was also run during the restricted time period 1995–2009 corresponding to the timing of the fish surveys with a significant decline in oxygen at a rate of 0.70 μmol O_2_/kg per year (Table S2). Analogous models assessing changes in temperature and salinity at the same CalCOFI stations from 1990 to 2019 found no significant temporal trend (Table S3).

**TABLE 1:** Results of linear regression: Oxygen concentration in September, October and November from 1990–2019 at CalCOFI line 83.3 station 42, line 86.7 station 45, and line 83.3 station 51 as a function of year as a continuous variable and depth interval (*z*) as a categorical variable. Depth intervals of 50 m spanned the depth range from 50 to 300 m

	Estimate	Std. Error	*t*‐value	*p*‐value
(Intercept)	1271.74	257.85	4.93	1.20E−06
year	−0.53	0.13	−4.15	4.15E−05
*z* = 100 m	−59.21	2.81	−21.09	<2E−16
*z* = 150	−93.71	4.09	−22.90	<2E−16
*z* = 200	−124.82	3.66	−34.14	<2E−16
*z* = 250	−139.77	4.35	−32.12	<2E−16
*z* = 300	−154.19	4.35	−35.43	<2E−16

There is a wide range of definitions for environmental hypoxia in the literature (Hofmann et al., 2011). For the purposes of this study, we defined oxygen concentrations in the ocean as mildly hypoxic at concentrations below 107 μmol O_2_/kg seawater (≈2.45 mL O_2_/L, Committee on Environment & Natural Resources, 2010, Hofmann et al., 2011) and hypoxic below 61 μmol O_2_/kg (≈1.4 mL O_2_/L, Hofmann et al., 2011, Middleburg & Levin, 2009). In this study, water at the 150‐m depth interval, corresponding to surveys conducted on Footprint reef, began to fall into the mildly hypoxic category by 2004 (Figure 3). Oxygen concentrations at depths of 200 m and below were mildly hypoxic throughout the time series. Oxygen concentrations in the 250‐m depth category became hypoxic in 2005. Finally, oxygen concentrations at the 300‐m depth category, corresponding to the depth of Piggy Bank reef, were consistently hypoxic throughout the time series (Figure 3).

There were 60 distinct combinations of fish taxonomic group and life stage that met the minimum observation thresholds for inclusion in the analysis. A Poisson or quasi‐Poisson model was developed for each taxon and life stage combination, representing 44 taxonomic groups, and including 16 YOY stages and 44 non‐YOY stages (Table 2, Table S4). For some taxa, only one of the two life stages was observed frequently enough to be included in this analysis. The model results for all 60 combinations of taxonomic group and life stage are presented in the supplementary materials (Table S4).

**TABLE 2:** Results of Poisson or quasi‐Poisson models for each combination of fish species and life stage with a significant interaction between year and depth. Species with modelled peak distributions that fall outside the depth range in which that species was observed are not included in this table. The results of all 60 Poisson or quasi‐Poisson models are provided in Table S4

Scientific Name	Life stage	Model	*β*_0_	*β*_depth_	*p*‐value	*β*_year_	*p*‐value	*β*_depth_^2^	*p*‐value	*β*_year*depth_	*p*‐value
*Rhinogobiops nicholsii*	Adult	QP	−1.49E+03	2.30E+01	4.16E−02	7.43E−01	5.52E−03	−7.98E−04	3.09E−01	−1.15E−02	4.06E−02
*Rhinogobiops nicholsii*	YOY	QP	−1.88E+03	2.67E+01	1.73E−03	9.24E−01	1.56E−05	−5.37E−03	2.90E−08	−1.30E−02	2.20E−03
*Sebastes mystinus*	Adult	QP	−2.29E+03	4.79E+01	4.02E−02	1.12E+00	3.45E−02	−2.09E−02	1.22E−01	−2.28E−02	4.01E−02
*Sebastes mystinus*	YOY	P	−1.33E+04	2.90E+02	2.57E−04	6.28E+00	6.43E−04	−2.69E−01	4.25E−06	−1.31E−01	3.70E−04
*Chromis punctipinnis*	Adult	QP	−1.96E+04	4.27E+02	9.78E−37	9.58E+00	2.15E−35	−1.77E−01	5.64E−32	−2.05E−01	1.41E−36
*Chromis punctipinnis*	YOY	P	−3.85E+03	8.61E+01	2.45E−06	1.93E+00	5.94E−06	−9.52E−04	4.27E−01	−4.31E−02	2.17E−06
*Oxylebius pictus*	YOY	P	−4.06E+03	8.85E+01	2.00E−03	2.03E+00	2.16E−03	−4.54E−04	5.02E−01	−4.43E−02	1.97E−03
*Sebastes caurinus*	Adult	QP	−4.98E+03	1.03E+02	2.16E−17	2.47E+00	2.69E−17	−6.48E−03	9.39E−09	−5.12E−02	2.54E−17
*Sebastes carnatus*	Adult	QP	−4.16E+03	8.89E+01	2.32E−03	2.06E+00	1.65E−03	−1.84E−02	2.66E−01	−4.36E−02	1.76E−03
*Sebastes semicinctus*	Adult	P	−4.88E+02	3.78E+00	9.31E−07	2.35E−01	9.20E−08	−1.27E−03	1.13E−286	−1.75E−03	5.26E−06
*Sebastes semicinctus*	YOY	P	1.35E+02	−2.97E+00	4.17E−31	−6.98E−02	4.92E−18	−8.53E−04	3.54E−221	1.54E−03	2.09E−33
*Sebastes ovalis*	Adult	QP	−1.26E+03	1.22E+01	2.94E−03	6.19E−01	1.63E−02	−1.16E−03	1.27E−06	−5.94E−03	3.46E−03
*Phanerodon furcatus*	Adult	QP	−6.65E+03	1.53E+02	1.87E−02	3.04E+00	3.82E−02	−2.47E−01	2.86E−05	−6.45E−02	3.71E−02
*Sebastes simulator*	Adult	P	−4.11E+02	2.02E+00	6.57E−14	2.00E−01	1.18E−11	−1.10E−04	1.09E−56	−9.78E−04	2.64E−13
*Sebastes wilsoni*	Adult	QP	−7.74E+02	7.40E+00	4.23E−03	3.80E−01	1.65E−02	−8.75E−04	6.79E−14	−3.59E−03	5.24E−03
*Rhacochilus toxotes*	Adult	P	4.34E+03	−9.30E+01	3.57E−02	−2.17E+00	3.52E−02	−3.26E−03	4.33E−01	4.65E−02	3.64E−02
*Sebastes spp*.	Adult	P	1.94E+02	1.13E+00	2.81E−02	−1.02E−01	5.89E−03	−3.10E−04	9.18E−75	−5.20E−04	4.34E−02
*Oxyjulis californica*	Adult	QP	−8.96E+03	2.08E+02	1.07E−12	4.47E+00	3.09E−11	−8.53E−03	3.50E−14	−1.04E−01	1.40E−12
*Sebastes zacentrus*	Adult	P	−1.01E+04	4.39E+01	5.78E−07	4.90E+00	6.74E−07	−5.19E−03	4.36E−10	−2.07E−02	9.91E−07
*Semicossyphus pulcher*	Adult	QP	−1.97E+03	4.01E+01	1.74E−02	9.44E−01	1.36E−02	−3.10E−02	2.16E−03	−1.84E−02	2.21E−02
*Sebastes hopkinsi*	Adult	P	3.22E+02	−4.69E+00	0.00E+00	−1.63E−01	0.00E+00	−7.78E−04	0.00E+00	2.41E−03	0.00E+00
*Sebastes constellatus*	Adult	QP	−3.13E+02	2.42E+00	3.55E−02	1.53E−01	1.47E−02	−2.05E−04	3.03E−07	−1.19E−03	3.81E−02
*Embiotocidae*	Adult	QP	1.16E+04	−2.39E+02	5.48E−08	−6.03E+00	1.91E−09	−2.14E−01	1.69E−05	1.29E−01	1.74E−09

**FIGURE 4:**
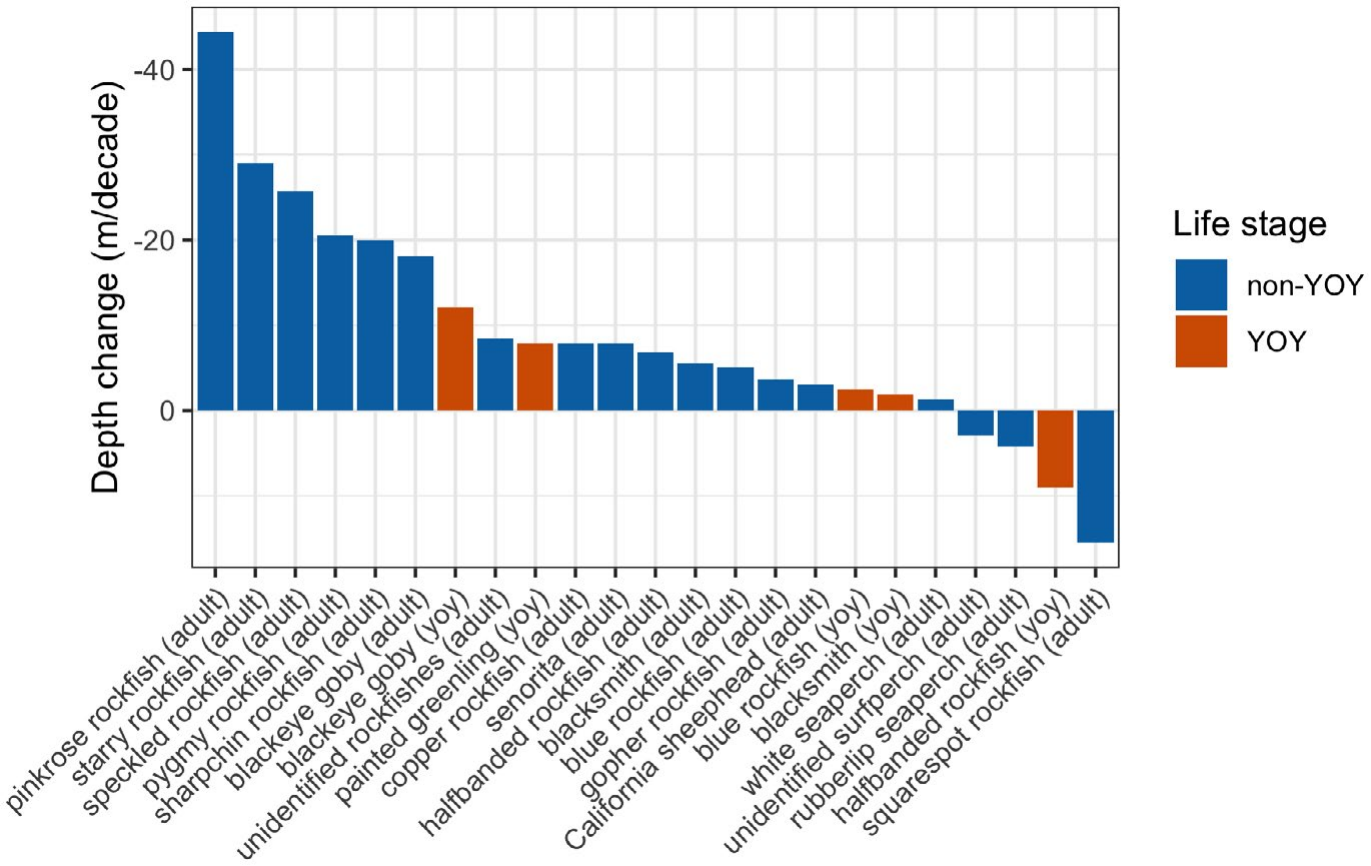
Depth change (m/decade) of peak distribution estimated by Poisson or quasi‐Poisson models for each fish species and life stage. Bar plot shows only species with a significant interaction term between depth and year that have at least one modelled annual peak abundance that occurred within the range of depths observed in this study. Negative depth changes indicate fish distributions that are occurring shallower through time, and positive depth changes indicate fish distributions that are occurring deeper through time

Species‐ and life stage‐specific changes in peak depth distribution of fish over time were examined using the interaction term between depth and year in the Poisson and quasi‐Poisson models. Out of the 60 modelled combinations of fish taxonomic group and life stage, 27 of the models had a statistically significant interaction between depth and year. In four of these fish types, there was no model‐estimated peak in depth distribution within the observed depth range (Figure 4; Table 2). In the remaining 23 fish types with a significant interaction between depth and year, the depth of peak fish abundance became shallower over time in 19 of the fish types, consisting of 15 non‐YOY taxa and four YOY taxa (Figure 4; Table 2). The depth of peak fish abundance became deeper over time in only four of the fish types, including one YOY taxon (Figure 4).

**FIGURE 5:**
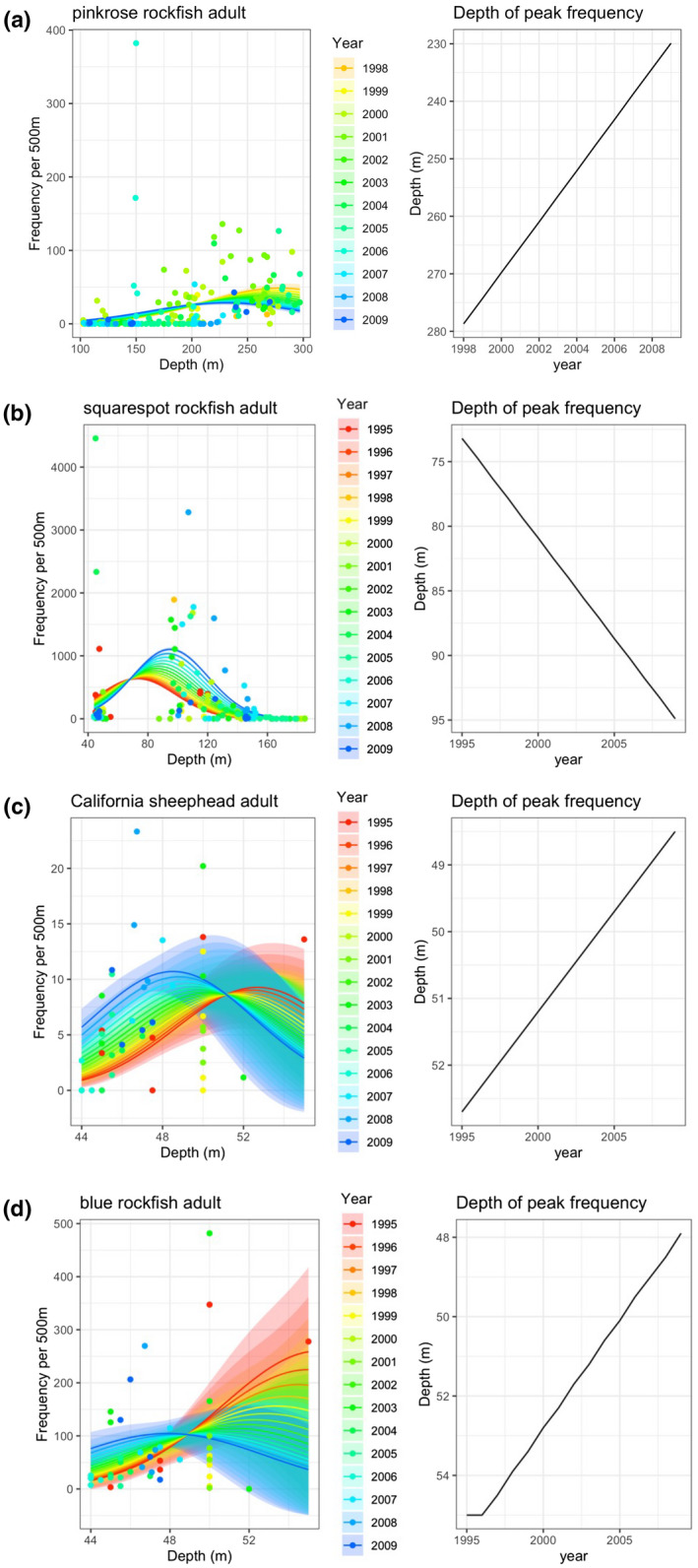
Left panels show the Poisson or quasi‐Poisson model estimates (lines) and 95% confidence intervals (polygons) of fish density (individuals per 500 m transect) as a function of depth in metres for a given species and life stage. Points indicate observed fish densities. Line, polygon and point colours correspond to the year of the observation or input into the model to provide a model estimate. Right panels show model‐estimated depth of peak fish density as a function of year. Figure a: adult pinkrose rockfish (*Sebastes simulator*), b: adult squarespot rockfish (*Sebastes hopkinsi*), c: adult sheephead (*Semicossyphus pulcher*) and d: adult blue rockfish (*Sebastes mystinus*)

Among the 23 fish types with a significant change in peak depth distribution over time, the average model‐estimated change in depth was −8.7 m/decade, where the negative sign indicates that the distributions overall are shifting to shallower habitat. Non‐YOY pinkrose rockfish (*Sebastes simulator*; Figure 2c) exhibited the largest shift into shallower habitat, with a change in peak distribution from 279 m to 230 m over a 12‐year observation period (Figure 5a). Non‐YOY squarespot rockfish (*Sebastes hopkinsi*; Figure 2a) exhibited the largest estimated shift into deeper habitat, with a change in peak distribution from 76 to 95 m over the 15‐year observation period (Figure 5b). Notably, and unlike the other species in this analysis, modelled squarespot rockfish peak distributions overlapped with a gap in our surveyed depth distribution corresponding to the bathymetric slope between the Anacapa Passage and the Footprint reefs (Figure 5b).

Many species that are valuable to commercial and recreational fisheries exhibited statistically significant changes in peak depth distribution. Non‐YOY California sheephead (*Semicossyphus pulcher*), one of the most valuable commercial fishery species in the region (California FWS, 2020), demonstrated a distribution shift to shallower water of −3.0 m/decade (Figure 5c). Blue rockfish (*Sebastes mystinus*; Figure 2b), one of the most valuable recreational fisheries in the region (RecFIN, 2020), demonstrated a distribution shift to shallower water of −5.1 m/decade for non‐YOY (Figure 5d) and −2.4 m/decade for YOY life stages (Figure 4).

## 4 DISCUSSION

Oceanographic measurements indicate that DO has declined over the previous three decades in the shelf waters of the Southern California Bight. The rate of DO decline of 0.53 μmol O_2_/kg per year is consistent with trends examined in the California Current and Northeast Pacific in previous studies (summarized in Ren et al., 2018). During the time period 1995–2009, about one third (19 out of 60) of the fish types observed on rocky reefs exhibited a significant shift in their peak distribution to shallower habitat. Peak distribution shifts to shallower habitats are occurring at variable rates, ranging from −1.3 m/decade to −44.3 m/decade vertically (Figure 4), depending on the fish species and life stage. A much smaller fraction of fish types (4 out of 60) exhibited a significant shift to deeper habitats. As anthropogenic warming and ocean deoxygenation intensify, the rapid redistribution of marine species to more suitable habitats and the extirpation of populations that are unable to relocate will have unprecedented consequences on marine ecosystem structure and the provisioning of ecosystem resources (Breitburg et al., 2018; Keeling et al., 2009; Levin et al., 2009).

The distributions of fish and invertebrate species, including species with high commercial or recreational fishery value, have been associated with DO in previous studies (Chan et al., 2008; Gallo, Beckwith, et al., 2020; Gallo, Hardy, et al., 2020; Keller et al., 2010, 2015, 2017). However, it is difficult to disentangle ecosystem response to persistent or seasonal low DO due to local bathymetry or local‐scale oceanographic processes from multidecadal trends in DO stemming from either natural climate oscillations or anthropogenic climate change when surveys occur only once or sporadically. Our results uniquely demonstrate a change in in situ distribution over a 15‐year period concurrent with an environmental decline in oxygen. This study leverages a rich data set documenting distribution shifts across rocky reefs ranging from 44 to 365 m deep but spanning a geographic distance of only ~10 km. The geographic proximity of the three reefs limits the effects of other external environmental factors. Repeated sampling with consistent methodologies over a 15‐year time period provided the analytical power to observe shoaling in peak reef fish distributions corresponding to climate‐driven deoxygenation. This underscores the value of long‐term continuous ecological monitoring programs for understanding the far‐reaching impacts of anthropogenic and environmental change.

Reef fish at the deepest site, Piggy Bank, and over time at the mid‐depth site, Footprint, were exposed to DO concentrations below levels found to have impacts on metabolism, swimming behaviour and predation in laboratory studies of several California rockfish species (Davis et al., 2018; Flannery, 2018). The observed shift in peak distributions of fishes to shallower depths may be explained by emigration to areas with higher DO concentrations, as supported by behavioural responses to hypoxia in laboratory settings (Davis et al., 2018; Flannery, 2018). Given the position of these reefs along the steep bathymetric gradient, more oxygen‐rich waters at shallower depths are easily accessible. There may also be an effect of increased predation mortality in hypoxic waters, as demonstrated by increased predation on juvenile rockfish by cabezon (*Scorpaenichthys marmoratus*) under hypoxic conditions in the laboratory (Davis et al., 2018).

Most of the fish life stages that demonstrated a significant depth shift over time were non‐YOYs. Only five YOY species exhibited a depth change, with four species shifting shallower in the water column and one species (halfbanded rockfish, *Sebastes semicinctus*) shifting deeper (Figure 4). YOY fishes at these natural reefs preferentially recruit to shallower habitats, then exhibit an ontological shift to deeper habitats (Love et al., 2009; Lowe et al., 2009), whereas non‐YOY fish density peaks in the 150–200 m depth range (Love et al., 2019). Given the depth ranges that were surveyed, this study is well designed to capture changes in depth distribution for non‐YOY fishes; however, additional surveys in the 0–50 m range may be necessary to assess depth shifts for YOY life stages.

While signals of ocean deoxygenation driven by anthropogenic climate change have emerged globally (Stramma et al., 2012; Keeling et al., 2010; Helm et al., 2011), natural climate oscillations are superimposed on these trends and can confound mechanistic explanations. The visual surveys in this study coincided with a period of anomalously low DO concentrations in the California Current region (Bograd et al., 2008; McClatchie et al., 2010), likely linked to a shallow thermocline characterized by a negative (cool) phase of the Pacific Decadal Oscillation (PDO) (Deutsch et al., 2011; Stramma et al., 2019). The rapid rates of change in depth distribution estimated for the rocky reef fish species in this study occurred during a period of rapid oxygen decline; future decreases in DO driven by anthropogenic climate change may be more gradual. Additionally, while historical declines in DO provide some foresight into ecosystem response to impending anthropogenic climate change, other environmental factors such as nutrient availability will respond differently to natural and anthropogenic climate variability. For example, negative phases of the PDO are linked to colder and more productive environments (Deutsch et al., 2011; Stramma et al., 2019), although not necessarily in the California Current System (Di Lorenzo et al., 2008), whereas anthropogenic warming is projected to correspond to a decrease in nutrient availability (Behrenfeld et al., 2006; Polovina et al., 2008). Further study is required to tease apart the relative impacts of both natural and anthropogenic climate processes and predict future changes in fish distribution.

Fishing regulations and non‐linear population dynamics also have the potential to cause changes in fish distribution over time. The removal of living resources was prohibited starting in 2003 in the Footprint State Marine Reserve in response to the Marine Life Protection Act of 1999 (California Fish and Game Code Sections 2850–2863). This State Marine Reserve overlaps with most of our study area, including about half of the surveys in Anacapa Passage and all of the Footprint and Piggy Bank survey sites. ROV surveys monitoring the first 5 years after the establishment of nearby Channel Islands state marine reserves show higher abundances of rocky reef fishes inside the no‐take reserves relative to paired sites outside the reserves (Karpov et al., 2012). Furthermore, fish recruitment, especially in rockfish species, is not consistent over time (e.g. Zabel et al., 2011). For example, a known pulse in juvenile rockfish recruitment occurred in 1999, and this cohort was followed at natural reefs and offshore oil and gas platforms until at least 2004 (Love et al., 2006; Meyer‐Gutbrod et al., 2019). Although this study does not critically examine changes in abundance over time, but rather changes in peak depth distribution, temporal patterns in cohort size stemming from either changes in fishing pressure or high recruitment years may lead to confounding patterns in ontological depth preferences. Further analysis examining fish size/age distribution and anomalous cohort size over time would shed light on these potential mechanisms of variation in depth distribution.

There were several limitations in this study that may inform the planning of future climate change impact research on marine ecosystems. Most importantly, the estimates of changes in vertical distribution over time will be improved with a longer time series of consistent sampling. Climate impacts are best assessed at the multidecadal scale, and the 15‐year time series presented here is just long enough to provide a meaningful assessment of inter‐annual trends in deep water rocky reef ecosystems. Previous work on the impacts of low DO on the distribution and diversity of marine organisms has consistently been limited by the lack of repeated sampling of the same habitats through time. One third of the fish species and life stage combinations tested in this study significantly changed in depth distribution over time; however, shifts in depth distribution may be resolvable for more of the study species if surveys are conducted over a longer time period.

In addition to the benefits of a longer time series, future studies would benefit from an expansion of depth ranges, especially up to the shallowest reefs when possible. Some species were excluded from the analysis because their model‐estimated peak distribution occurred outside the surveyed depth range of 44 to 365 m. Surveys of a shallower reef in the network would enable the inclusion of more species; however, reef networks that span such a large depth range are rare.

Water quality measurements were not collected concurrently with the visual surveys over the rocky reef system in this study. The comparison of fish distribution change with oxygen was enabled here by the consistent and long‐term sampling of oxygen concentration and temperature at three CalCOFI stations nearby; however, similar surveys could be improved with the collection of in situ oceanographic measurements. Precise DO data collected at each transect would be ideal for building mechanistic models of the effects of DO on fish population and community dynamics.

Surveys that include in situ oxygen concentration sampling would also be useful for identifying fish response to seasonal patterns in DO. While all fish surveys and CalCOFI station sampling included in this study occurred within a 64‐day seasonal window over the 15‐year time series (Figure S1), fish survey effort was not high enough to parse out potential seasonal variability within that survey window. The fall period examined in this study is concurrent with a seasonal shift from summertime upwelling‐driven hypoxia to higher bottom‐water DO levels in the winter, although these oscillations are less pronounced in the southern portion of the California Current such as the Santa Barbara Channel (Peterson et al., 2013). Most rockfish species, however, have small home ranges and high site fidelity with limited seasonal movements (Green et al., 2014, Jorgensen et al., 2006, Tolimieri et al., 2009). Although observations are limited, known seasonal movements of species such as copper and blue rockfishes are small in scale and occur over the summer, and therefore are less likely to impact our study (Matthews, 1990).

It is essential to document and predict distribution changes in fish species that are actively managed to monitor sustainable fishing practices and promote stock recovery. Among the fish species that exhibited a statistically significant distribution shift into shallower waters, California sheephead and copper rockfish (*Sebastes caurinus*) are both commercially valuable with >60,000 lbs each landed in the Santa Barbara Channel region in 2019 (California FWS, 2020). Blue rockfish, copper rockfish, rosy rockfish (*Sebastes rosaceus*) and starry rockfish (*Sebastes constellatus*) are all valuable to recreational fishers, with more than 25,000 individual landings documented for each of these species in the Santa Barbara Channel region in 2019 (RecFIN, 2020). The 33 fish species included in the analysis that did not exhibit a significant shift in depth distribution over time (Table S4) may be less sensitive to changes in oxygen or have developed adaptations to improve their fitness in lower oxygen environments. Vertical movements in response to environmental variables may occur slowly for some species, and longer sampling time series could be required to resolve significant distribution shifts. This study is most appropriate for detecting distribution shifts in species that exhibit limited daily and seasonal movement; higher resolution detections, perhaps using acoustic tags, would be more appropriate for detecting vertical distribution shifts in more active species.

The identification of hypoxic environments, tracking their spatial and temporal dynamics, and predicting the response of fish species to this environmental degradation is critical to supporting meaningful fishery management. Oxygen stress will emerge in nearly half of the global no‐take marine protected areas by 2050 under the business‐as‐usual climate projection RCP8.5 (Bruno et al., 2018). Hypoxic conditions may result in the degradation of 55% and complete loss of 18% of the available demersal habitat within the established Cowcod Conservation Area in the Northeast Pacific by 2030, significantly undermining the benefit of this management initiative (McClatchie et al., 2010). Hypoxic conditions cause fish to aggregate in high densities in refuge habitats at the boundaries of low oxygen regions, which creates opportunities for increased exploitation by predators and commercial and recreational fishers (Breitburg et al., 2018; Eby & Crowder, 2002; McCormick & Levin, 2017; Roman et al., 2012; Stramma et al., 2012). Shifting distributions of marine fish and invertebrates in search of oxygen refugia have the potential to further complicate fishery management by increasing the risk of bycatch (Craig & Bosman, 2013).

Declining oxygen will exceed the range of natural variability in most of the global ocean by 2052 (Henson et al., 2017). Climate models predict a continued decline of up to 7% in global oceanic DO concentrations in the next century (Bopp et al., 2013; Keeling et al., 2010). Monitoring the formation and severity of these low oxygen habitats and the ecosystem response will be a critical component to the effective placement of marine protected areas and the regulation of recreational and commercial fisheries. This study demonstrates significant changes in the depth distribution of rocky reef fish species over a 15‐year time period and underscores the need for fisheries management that is responsive to variable, and potentially unprecedented, environmental conditions.

## Supporting information

Fig S1

Table S1

Table S3

Table S4

Supplementary Material

## Data Availability

The fish survey data that support the findings of this study are openly available in the ‘Santa Barbara Channel fish surveys at deep reefs: Footprint, Piggy Bank, Anacapa Passage’ repository hosted by the Environmental Data Initiative at https://doi.org/10.6073/pasta/59d44ccc0d08bb8735a564aca91e5009. The oceanographic data that support the findings of this study are openly available in the ‘CalCOFI Hydrographic Database’ at https://calcofi.org/ccdata.html
