# Supplementary figures and images for: Moving on up: Vertical distribution shifts in rocky reef fish species during climate‐driven decline in dissolved oxygen from 1995 to 2009
Source: Glob Chang Biol. 2021 Sep 16;27(23):6280–93. doi: 10.1111/gcb.15821 (PMC9290838; doi:10.1111/gcb.15821)

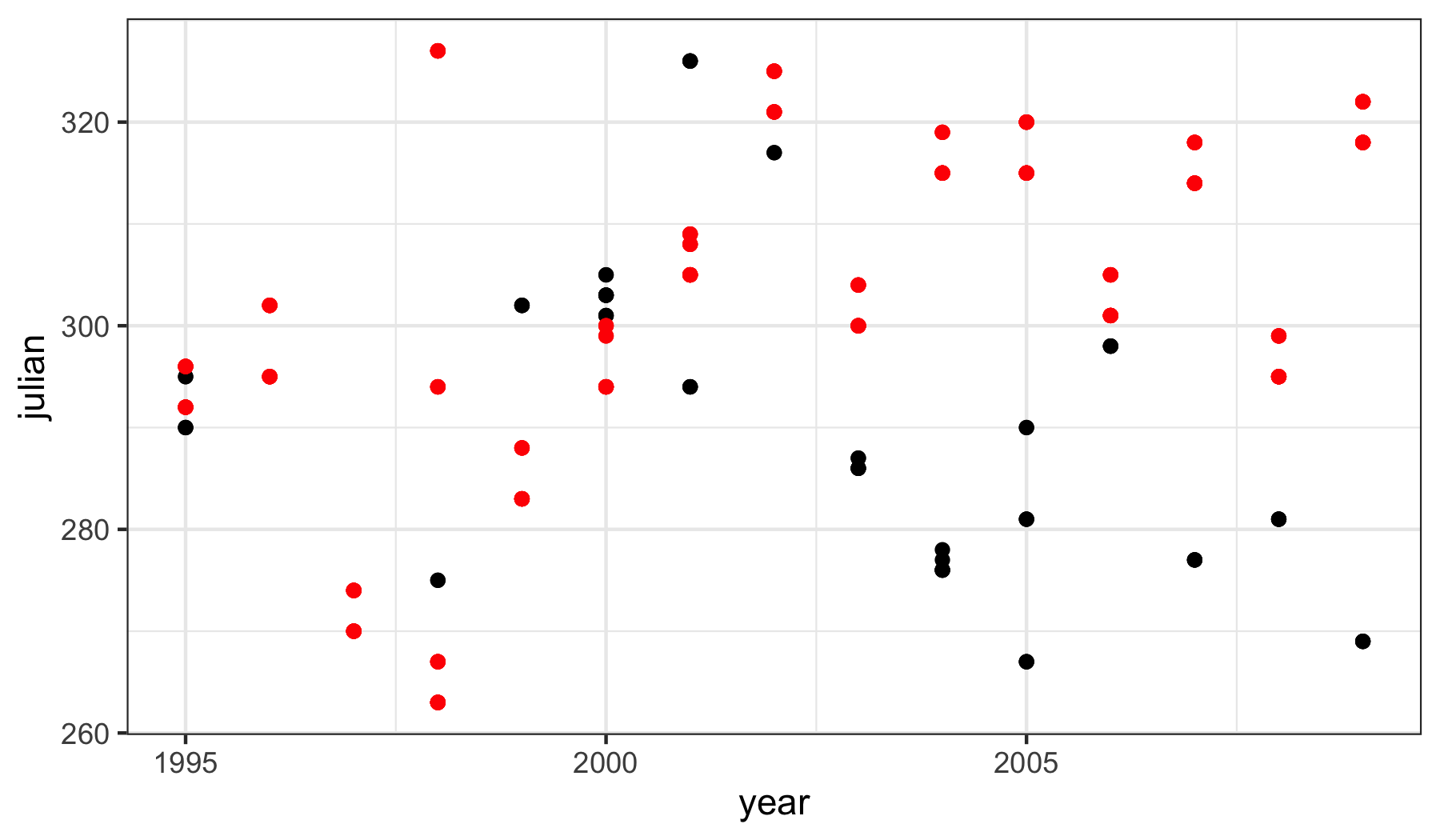

Supplement: Supplementary file 1 — Fig S1 [file GCB-27-6280-s001.png]
