# Supplementary material for: Moving on up: Vertical distribution shifts in rocky reef fish species during climate‐driven decline in dissolved oxygen from 1995 to 2009
Source: Glob Chang Biol. 2021 Sep 16;27(23):6280–93. doi: 10.1111/gcb.15821 (PMC9290838; doi:10.1111/gcb.15821)
Supplement: Supplementary file 5 — Supplementary Material [file GCB-27-6280-s003.docx]

SUPPORTING INFORMATION

Moving on up: Vertical distribution shifts in rocky reef fish species during climate-driven decline in dissolved oxygen from 1995-2009

Erin Meyer-Gutbrod^1^, Li Kui^2^, Robert Miller^2^, Mary Nishimoto^2^, Linda Snook^2^, Milton Love^2^

1. School of the Earth, Ocean and Environment; University of South Carolina; emgutbrod@seoe.sc.edu
2. Marine Science Institute; University of California, Santa Barbara

SUPPORTING INFORMATION

Table S1: YOY total length cutoff in centimeters for each species included in this study

Table S2: Results of linear regression: Oxygen concentration in September, October and November from 1995-2009 at CalCOFI line 83.3 station 42, line 86.7 station 45, and line 83.3 station 51 as a function of year as a continuous variable and depth interval (z) as a categorical variable. Depth intervals of 50 m spanned the depth range from 50-300m.

|  | **Estimate** | **Std. Error** | **t-value** | **p-value** |
| --- | --- | --- | --- | --- |
| **(Intercept)** | 1600.85 | 675.19 | 2.37 | 1.87E-02 |
| **year** | -0.70 | 0.34 | -2.07 | 3.94E-02 |
| **z = 100 m** | -61.91 | 3.72 | -16.66 | <2E-16 |
| **z = 150** | -94.79 | 5.76 | -16.47 | <2E-16 |
| **z = 200** | -124.47 | 4.74 | -26.27 | <2E-16 |
| **z = 250** | -140.28 | 6.06 | -23.14 | <2E-16 |
| **z = 300** | -153.83 | 6.06 | -25.38 | <2E-16 |

Table S3: Results of 2 linear regression models examining changes in 1) Temperature and 2) Salinity in September, October and November from 1990-2019 at CalCOFI line 83.3 station 42, line 86.7 station 45, and line 83.3 station 51 as a function of year as a continuous variable and depth interval (z) as a categorical variable. Depth intervals of 50 m spanned the depth range from 50-300m.

Table S4: Results of poisson or quasipoisson models for each combination of fish species and life stage.


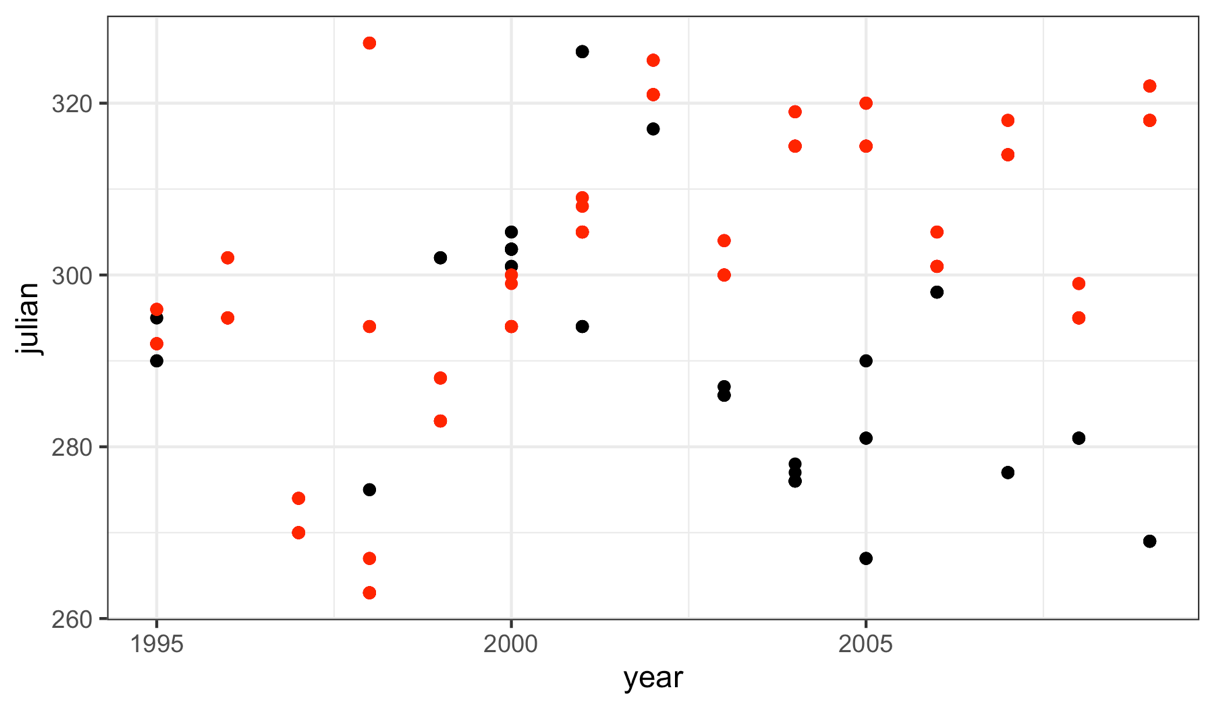


Figure S1: Interannual variability of sampling dates for ROV fish surveys (black points) and CalCOFI cruise sampling at line 83.3 station 42, line 86.7 station 45, and line 83.3 station 51 (red points). Dates on the y-axis are expressed as Julian days.
